# Complete Genome Sequence of a New Strain of Sida Golden Mosaic Buckup Virus from Florida, USA

**DOI:** 10.1128/MRA.01115-19

**Published:** 2020-01-09

**Authors:** Zafar Iqbal, Salma Arous, J. E. Polston

**Affiliations:** a Department of Plant Pathology, University of Florida, Gainesville, Florida, USA; b Higher Institute of Biotechnology Sidi Thabet, University of Manouba, Sidi Thabet, Tunisia; Portland State University

## Abstract

The complete genome sequence of a bipartite begomovirus found in a Sida sp. plant growing in Bradenton, FL, was determined. The virus is a new strain of Sida golden mosaic Buckup virus (family Geminiviridae, genus Begomovirus). This is the first report of this virus in the United States and the first report outside Jamaica.

## ANNOUNCEMENT

*Sida* is a genus of plants (family Malvaceae) that is distributed around the globe, primarily in the tropics and subtropics (1). *Sida* spp. are perennial hosts of many begomoviruses (genus Begomovirus, family *Geminiviridae*) and provide an environment for their evolution (2). Begomoviruses have single-stranded circular DNA genomes with one or two genome components, designated DNA-A and DNA-B (3).

In 1997, leaves from a *Sida* sp. plant that exhibited a golden mosaic were collected from Bradenton, FL, and stored at −80°C. DNA was extracted from the leaves (4), and full-length DNA-A and DNA-B genomes were amplified using phi29 and random primers. A partial genome sequence was obtained from each DNA by first using degenerate primers (5), followed by primer walking using primers designed from the obtained sequences (6). Sequences were assembled using SeqMan v5.0 (DNAStar, USA). The sequence of DNA-A (GenBank accession number MK256739) was 2,611 nucleotides (nt) and had a GC content of 46.30%, and DNA-B (GenBank accession number MK256740) was 2,579 nt and had a GC content of 44%. These sequences are cognate components based on their common regions (110 nt), which shared 100% sequence identity. Clones of DNA-A and DNA-B were infectious when inoculated into Nicotiana benthamiana (7). The genome organization was typical of New World bipartite begomoviruses, encoding five predicted open reading frames (ORFs) on DNA-A and two ORFs on DNA-B.

**FIG 1:**
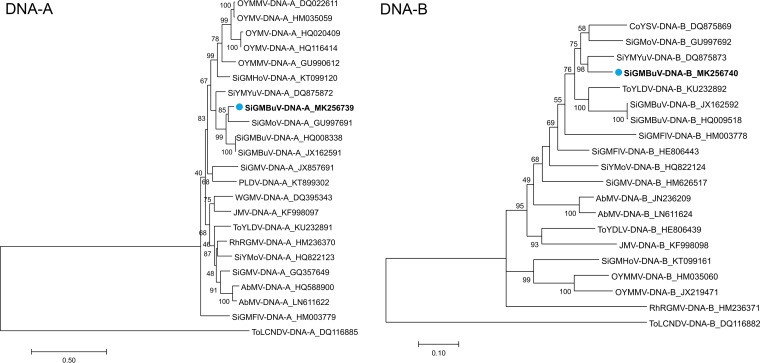
Phylogenetic tree of sequences of the begomoviruses with highest similarity to the DNA-A and DNA-B of SiGMBuV-Florida. The sequence identifiers include the virus acronym and NCBI accession number. The novel sequences of SiGMBuV-Florida are identified with a dot and bold font.

Pairwise nucleotide comparisons were conducted using SDT v1.2 (8). Sequences with the highest similarity to the DNA-A and DNA-B sequences were selected using BLASTn. Multiple-sequence alignment for phylogenetic analysis of DNA-A and DNA-B sequences was conducted using MUSCLE (9). Phylogenetic lineages and evolutionary histories were inferred using the maximum likelihood algorithm after selecting the general time-reversible (GTR+G) model (10); the decision to use this model was based on the Akaike information criterion (AIC) and Bayesian information criterion (BIC) in MEGA7 (11). Optimized phylogenetic dendrograms (1,000 bootstrap replicates) were constructed (Fig. 1) using MEGA7 (11). DNA-A had the highest identity score (93%) with sequences of Sida golden mosaic Buckup virus (SiGMBuV; GenBank accession numbers JX162591 and HQ008338). Similar to the Florida isolate, SiGMBuV was obtained from an unidentified *Sida* species from Jamaica (12). However, in phylogenetic analyses, DNA-A segregated and formed a separate clade (85% of bootstrap replicates) with Sida golden mottle virus (SiGMoV; accession number GU997691) isolated from Sida santaremensis in Florida (6). DNA-B had its highest identity score (89%) and grouped with Sida yellow mosaic Yucatan virus (SiYMYuV; accession number DQ875873) (98% of bootstrap replicates) isolated from Sida acuta in Yucatan, Mexico (13).

Recombinational analysis was performed using seven algorithms (Bootscan, Chimaera, GENECONV, MaxChi, RDP, SiScan, and 3Seq) in the RDP4 program (beta 4.97), using 51 virus sequences that had greater than 70% sequence identity with the new sequences (8). A recombination event was considered likely if detected by four or more methods (*P* value cutoff, 0.05). Three putative recombination events were detected in DNA-A, and one was detected in DNA-B. DNA-A (accession number MK256739) was a major parent of the DNA-As of SiYMYuV DNA-A (accession number DQ875872) and SiGMoV (accession number GU997691), according to RDP4 (14). These analyses were consistent with the grouping of DNA-A with SiYMYuV (accession number DQ875872).

These sequences represent a new strain of SiGMBuV and, according to approved nomenclature, should be referred to as SiGMBuV-Florida (15).

### Data availability.

Sequences were submitted to GenBank under accession numbers MK256739 (DNA-A) and MK256740 (DNA-B).
